# Systemic inflammation response index (SIRI) predicts prognosis in hepatocellular carcinoma patients

**DOI:** 10.18632/oncotarget.16865

**Published:** 2017-04-05

**Authors:** Litao Xu, Shulin Yu, Liping Zhuang, Peng Wang, Yehua Shen, Junhua Lin, Zhiqiang Meng

**Affiliations:** ^1^ Department of Integrative Oncology, Fudan University Shanghai Cancer Center, Shanghai 200032, China; ^2^ Department of Oncology, Shanghai Medical College, Fudan University, Shanghai 200032, China

**Keywords:** hepatocellular carcinoma, systemic inflammation response index, local therapy, systemic therapy, survival

## Abstract

The systemic inflammation response index (SIRI) is a useful tool for predicting prognosis in some types of cancer. In this retrospective study, we evaluated the efficacy of SIRI in predicting overall survival in hepatocellular carcinoma (HCC) patients following local or systemic therapy. A cutoff value of 1.05 was identified for SIRI using ROC analysis in a training patient cohort. In the validation cohort, survival analysis revealed that median overall survival was longer in HCC patients with SIRI scores < 1.05 than in those with scores ≥ 1.05. Cox analysis of the validation cohort demonstrated that SIRI was associated with overall survival and was more predictive of overall survival that the AFP level or Child-Pugh score. However, SIRI and Barcelona Clinic Liver Cancer (BCLC) stage were equally effective for predicting survival. In addition, HCC patients with BCLC stage C had higher SIRI scores and poorer overall survival. SIRI also correlated with liver function parameters. Thus SIRI may be a convenient, low cost and reliable tumor marker for predicting prognosis in HCC patients.

## INTRODUCTION

Hepatocellular carcinoma (HCC) is the fourth most common malignancy and the third most common cause of cancer-related death in China [1]. Surgical resection and liver transplantation are effective treatments for patients with early stage HCC, while transarterial chemoembolization (TACE), radiology, and sorafenib are the most commonly used treatments for patients with intermediate or advanced HCC at the time of diagnosis. However, useful prognostic tools for predicting HCC patient survival and assisting in tailoring therapies for patients who are at risk of worse outcomes currently do not exist.

Cancer-related inflammation can be considered the seventh hallmark of cancer [2]. Local immune response and systemic inflammation play important roles in cancer progression and patient survival [3]. White cell counts and ratios, including neutrophil, lymphocyte, and monocyte counts and neutrophil/lymphocyte ratio (NLR) and platelet/lymphocyte ratio (PLR), as well as C-reactive protein levels and the Glasgow prognostic score, have been identified as potential prognostic tools for many types of cancer [4–8]. However, some studies have suggested that neither NLR nor C-reactive protein levels are accurate prognostic markers for cancer progression or overall survival [5, 9].

In this study, we evaluated the ability of the systemic inflammation response index (SIRI), which predicted survival in pancreatic cancer patients [10], to predict overall survival in HCC patients following local therapy.

## RESULTS

### Patient characteristics

As shown in Table 1, a total of 351 HCC patients were enrolled in this study; 168 patients were assigned to the training cohort and 183 were assigned to the validation cohort. All clinical variables were measured at baseline, which was defined as 3 days before treatment, and all patients were diagnosed with HCC at our hospital. Clinical characteristics were similar between the training and validation cohort patients and between the Portal Vein Tumor Thrombus (PVTT) group and no PVTT group in the training cohort (all *P* > 0.05). Receiver operating characteristic (ROC) analysis was used to identify 1.05 × 10^9^ as the optimum cutoff value for SIRI in the training cohort, and this cutoff value was used for all subsequent analyses (Supplementary Figure 1).

**Table 1:** Clinical characteristics of HCC patients in the training and validation cohorts

Variable	Training Set, *n* = 168			Validation Set, *n* = 183	
	PVTT	No-PVTT	*P* value		*P’* value
No. of patients	47	121		183	
Age, years, Mean ± SD	53.9 ± 10.9	52.8 ± 12.5	0.396	53.7 ± 10.5	0.245
Gender			0.829		0.669
Male	41	104		155	
Female	6	17		28	
Hepatitis B, *n* (%)	38 (81)	103 (85)	0.498	156 (85)	0.733
Child-Pugh			0.759		0.665
A	42	110		163	
B	5	11		20	
AFP			0.204		0.861
< 200ng/ml	17	58		80	
≥ 200ng/ml	29	64		103	
Tumor size			0.000		0.803
≥ 5cm	36	0		41	
< 5cm	11	121		142	
TBIL (umol/L), mean ± SD	19.3 ± 17.8	15.0 ± 5.9	0.112	18.2 ± 12.1	0.166
DBIL (umol/L), mean ± SD	9.0 ± 11.2	6.4 ± 6.5	0.137	7.0 ± 7.2	0.735
ALT (IU/L), mean ± SD	49 ± 32.3	53.4 ± 35.6	0.466	46.8 ± 49.5	0.790
AST (IU/L), mean ± SD	75.1 ± 48.5	68.0 ± 52.1	0.422	61.1 ± 64.1	0.947
GGT (IU/L), mean ± SD	217.3 ± 164.5	196.7 ± 220.7	0.564	170.6 ± 191.7	0.792
LDH (IU/L), mean ± SD	273.2 ± 163.1	236.6 ± 141.4	0.152	237.9 ± 144.6	0.685
ALP (IU/L), mean ± SD	214.1 ± 287.5	131.4 ± 70.5	0.057	144.3 ± 110.4	0.996
ALB (IU/L), mean ± SD	38.9 ± 6.4	39.7 ± 4.8	0.391	39.1 ± 4.7	0.686

AFP, alpha fetoprotein; TBIL, total bilirubin; D-TBIL, direct bilirubin; ALT, alanine aminotransferase; AST, aspartate aminotransferase; GGT, glutamyl transferase; LDH, lactic dehydrogenase; ALP, alkaline phosphatase; ALB, albumin.

Clinical characteristics for validation cohort patients in the different SIRI groups are summarized in Supplementary Table 1. Barcelona Clinic Liver Cancer (BCLC) stage and alpha fetoprotein (AFP), aspartate aminotransferase (AST), gamma-glutamyl transferase (GGT), lactate dehydrogenase (LDH), alkaline phosphatase (ALP), and albumin (ALB) levels differed between the SIRI < 1.05 and SIRI ≥ 1.05 groups.

### Higher SIRI is associated with longer overall survival

**Figure 1:**
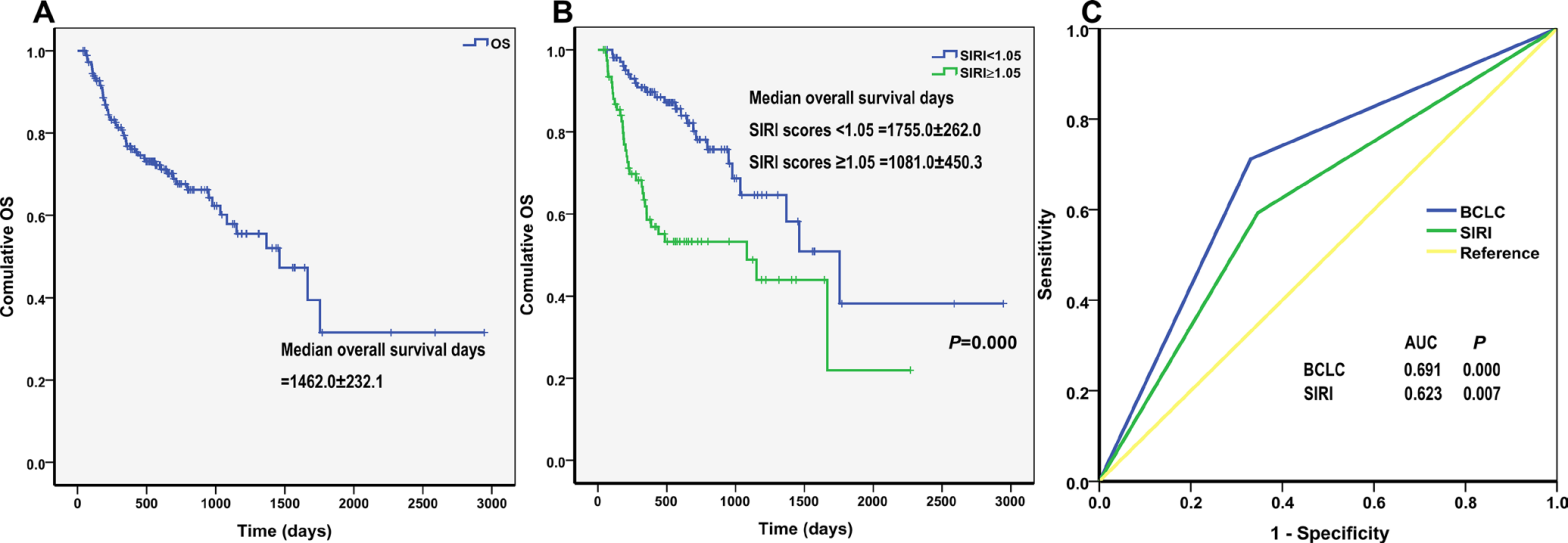
Kaplan-Meier curves for overall survival (OS) of (**A**) validation cohort and (**B**) different SIRI scores. The *P*-value was determined using the log-rank test. (**C**) Comparison of the sensitivity and specificity of survival prediction by AFP, Child-pugh, BCLC stage and SIRI through ROCs.

In the validation cohort, the 1-year, 3-year, and 5-year overall survival (OS) rates were 76.8%, 57.9%, and 31.5%, respectively, and the median survival time was 1462 days (Figure 1A). HCC patients with SIRI scores < 1.05, who had a median OS of 1755 days, survived longer than those with SIRI scores ≥ 1.05, who had a median OS of 1081 days (log rank, 14.604; *P* < 0.001) (Figure 1B).

Univariate analysis of the validation cohort indicated that AFP, Child-Pugh score, PVTT, metastasis, BCLC, PLR, and SIRI were associated with OS, while gender, Hepatitis B Virus (HBV), cirrhosis, tumor size, local therapy, sorafenib treatment, NLR, and lymphocyte-monocyte ratio (LMR) had no prognostic significance for OS (Table 2). Specifically, low SIRI scores were associated with longer OS (hazard ratio [HR], 2.663; 95% confidence interval [CI], 1.580–4.490; *P* < 0.001). Factors that were associated with OS in HCC patients in the univariate analyses were then included in a multivariate analysis with forward selection. As shown in Table 2, higher SIRI (HR = 2.111, 95% CI, 1.202–3.709, *P* = 0.009) and BCLC stage (HR = 5.207, 95% CI, 1.764–15.372, *P* = 0.003) were associated with poorer OS in HCC patients.

**Table 2:** Univariate and multivariate Cox regression analyses of SIRI associated with overall survival of HCC patients

Variables	Univariate analysis		Multivariate analysis	
	HR (95% CI)	*P* value	HR (95% CI)	*P* value
Gender: male vs female	0.633 (0.272–1.474)	0.289		
HBV: no vs yes	1.358 (0.687–2.683)	0.379		
Cirrhosis: no vs yes	0.750 (0.300–1.878)	0.539		
AFP: < 200 vs ≥ 200ng/ml	2.348 (1.344–4.100)	0.003	1.619 (0.900–2.914)	0.108
Child-pugh: A vs B	2.417 (1.278–4.572)	0.007	1.893 (0.961–3.727)	0.065
Tumor size > 5cm: no vs yes	1.705 (0.960–3.030)	0.069		
PVTT: no vs yes	3.216 (1.889–5.474)	0.000	0.853 (0.348–2.093)	0.729
Metastasis: no vs yes	2.440 (1.437–4.146)	0.001	0.649 (0.270–1.561)	0.334
BCLC: B vs C	5.046 (2.788–9.135)	0.000	5.207 (1.764–15.372)	0.003
Local therapy: no vs yes	0.687 (0.291–1.620)	0.391		
Sorafenib: no vs yes	1.529 (0.848–2.759)	0.158		
NLR: < 5 vs ≥ 5	1.844 (0.870–3.907)	0.110		
PLR: < 150 vs ≥ 150	2.176 (1.268–3.735)	0.005	1.363 (0.736–2.524)	0.324
LMR: < 3 vs ≥ 3	0.652 (0.387–1.098)	0.108		
SIRI: < 1.05 vs ≥ 1.05	2.663 (1.580–4.490)	0.000	2.111 (1.202–3.709)	0.009

### SIRI predicted overall survival

We then compared the sensitivity and specificity of SIRI for predicting overall survival in HCC patients to that of BCLC stage using ROCs. Both SIRI (AUC = 0.623, *P* = 0.007) and BCLC stage (AUC = 0.691, *P* < 0.001) predicted OS in HCC patients (Figure 1C). SIRI had a sensitivity of 59.3% and a specificity of 65.3% in predicting OS. Large, well-designed studies are needed to more thoroughly examine the prognostic roles of these factors in HCC patients.

### Comparison of SIRI in different HCC subgroups

**Figure 2:**
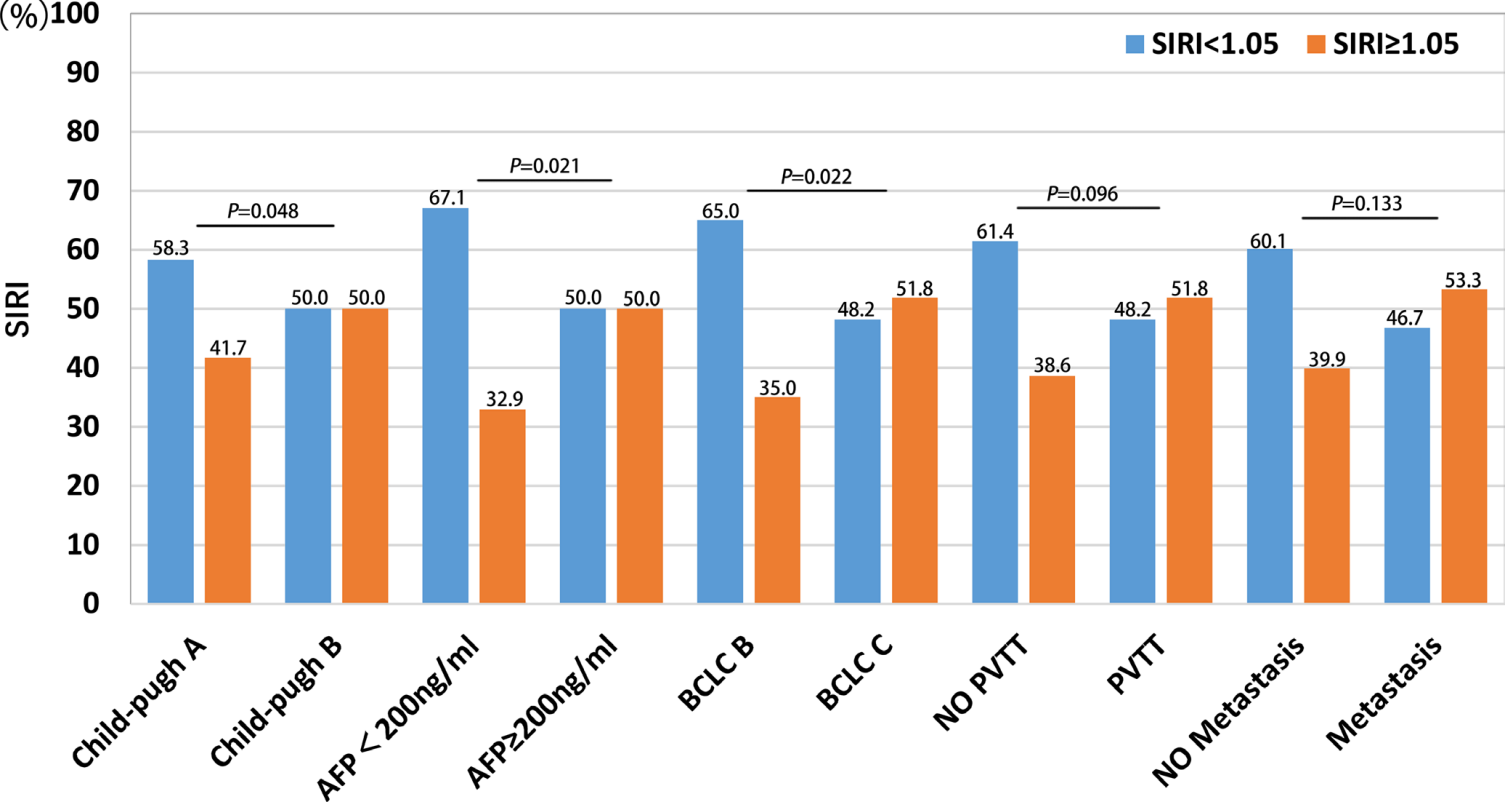
Comparison of SIRI in different HCC subgroups, including Child-pugh, AFP level, BCLC stage, PVTT and metastasis

Several clinical features, such as Child-Pugh score, AFP level, BCLC stage, PVTT, and metastasis, are risk factors associated with HCC survival. We compared associations between SIRI and these clinical features in different patient subgroups; the results are summarized in Figure 2. HCC patients with AFP ≥ 200 ng/ml and BCLC C stage had higher SIRI values than those with AFP ≥ 200 ng/ml and BCLC B stage. However, there were no associations between SIRI and Child-Pugh score, PVTT, or metastasis.

### SIRI was correlated with liver function

To investigate whether SIRI score was correlated with liver function, we assessed the following parameters: total bilirubin (TBIL), direct bilirubin (D-BIL), alanine aminotransferase (ALT), AST, GGT, LDH, ALP, ALB, prothrombintime activity percentage (PT), and international normalized ratio (INR) (Table 3). SIRI was correlated with all of the liver function parameters except for TBIL, PT, and INR.

**Table 3:** Correlation of SIRI and liver function parameters

Parameters	SIRI	
	Rank correlation coefficient (r)	*P* value
TBIL	0.073	0.145
D-TBIL	0.197	0.007
ALT	0.209	0.004
AST	0.321	0.000
GGT	0.223	0.000
LDH	0.318	0.000
ALP	0.279	0.000
ALB	−0.277	0.000
PT	−0.046	0.532
INR	−0.018	0.812

## DISCUSSION

Although HCC is one of the most common malignancies worldwide, few tumor markers with prognostic value in predicting HCC survival have been identified [11, 12]. It is therefore important to find convenient, low cost, and reliable tumor markers for predicting prognosis in HCC patients.

Systemic inflammation is an important promoter of the proliferation, invasion, and metastasis of malignant cells [13–15]. Higher neutrophil counts have been associated with poorer prognosis in many cancers [16]. Neutrophils in the peripheral blood or in the tumor microenvironment produce pro-angiogenic factors, such as vascular endothelial growth factor (VEGF), which stimulate tumor development and progression [17]. In addition, low lymphocyte counts weaken anti-cancer defenses, resulting in poorer prognosis [18]. Higher monocyte counts are associated with poorer prognosis in different cancers [19–21]. SIRI, a simple non-invasive prognostic marker based on peripheral neutrophil, monocyte, and lymphocyte counts, predicts survival in pancreatic adenocarcinoma patients after gemcitabine-based chemotherapy [10]. Although a different cut-off value for SIRI was used in this study, we demonstrated that SIRI was also an effective predictor of prognosis in patients with HCC. Furthermore, the predictive ability of SIRI was greater than that of NLR, PLR, LMR, and other conventional predictors such as AFP levels and Child-Pugh score. In addition, we found that SIRI was strongly associated with BCLC stage and AFP levels; HCC patients with AFP ≥ 200 ng/ml and BCLC C stage had higher SIRI values than those with AFP < 200 ng/ml and BCLC B stage. In addition, among the conventional indicators of liver injury, GGT, LDH, and ALP also reflected tumor burden in HCC patients. Here, we confirmed that SIRI was correlated with these parameters of liver function and with tumor burden.

Although our study demonstrated that SIRI was a useful tool for predicting prognosis in HCC, some limitations should be considered when interpreting the results. First, the retrospective nature of the study and the small patient sample size might have generated biases in the analysis. Second, SIRI and BCLC stage were equally effective in predicting overall survival in HCC patients. Third, some clinical features, such as portal hypertension, tumor size, and TNM stage, were not considered in this study. A well-designed, large prospective study is therefore needed to confirm the relationship identified here between SIRI and HCC prognosis.

In summary, SIRI may be a convenient, low-cost, and reliable marker for predicting prognosis in HCC patients. However, the mechanisms underlying the association between elevated SIRI and poorer prognosis in HCC patients require further investigation.

## MATERIALS AND METHODS

### Ethics statement

This study was approved by the Ethics Committee of Fudan University Shanghai Cancer Center. All procedures were performed in accordance with the ethical standards of our institutional research committee and with those of the 1964 Helsinki declaration and its later amendments (or comparable ethical standards). Written informed consent was obtained from each participant in accordance with institutional guidelines.

### Patients

We retrospectively enrolled HCC patients who were treated at Fudan University Shanghai Cancer Center between January 2006 and July 2013. Inclusion criteria were as follows: histologically confirmed HCC or clinical diagnosis based on dynamic imaging and an underlying chronic liver disease; a good performance status (ECOG level < 2); and favorable liver functions indicated by Child-Pugh class A or B. Exclusion criteria were as follows: history of another malignant disease within the last five years; cooccurrence of other lymphatic system disorders or malignant hematologic diseases; cooccurrence of renal and/or hepatic failure, acute coronary syndromes, valvular heart diseases, autoimmune thyroid diseases, or systematic inflammatory diseases.

### Study design

In this retrospective, single-center study, patients were assigned to either the training cohort or the validation cohort. All patients were first diagnosed with HCC in our hospital and had not received any previous treatment. Baseline patient characteristics, including demographics, tumor markers, routine blood test results, liver function parameters, and treatment history, were examined. The following variables were collected for analysis: age and gender; date of HCC diagnosis and date of death or last follow-up; presence of cirrhosis and ascites; main serological parameters, including TBIL, DBIL, ALT, AST, GGT, LDH, ALP, ALB, PT, and INR; and tumor characteristics and treatment history.

### SIRI

Previous studies have demonstrated that high peripheral neutrophil counts and NLR predict decreased survival in cancer patients [16]. Similarly, high numbers of circulating monocytes have been associated with increased tumor progression and poorer survival [20, 21]. The SIRI parameter was developed based on these results and has a higher prognostic value than other indicators of systemic inflammation in pancreatic cancer [10]. We therefore evaluated the efficacy of SIRI in predicting overall survival in HCC patients. SIRI was defined as previously reported [10]: SIRI = N × M/L, where N, M, and L are the pretreatment peripheral neutrophil, monocyte, and lymphocyte counts, respectively. However, the SIRI cutoff values used in this study, which were determined based on ROC analysis with the presence or absence of PVTT as a state variable in the training cohort, differed from those used in the previous study. The Youden index (sensitivity+specificity-1) was used to select a threshold for estimating sensitivity and specificity.

### Statistical analyses

Differences in patient characteristics between groups were evaluated using the Chi-squared test, Student's *t*-test, or the nonparametric Wilcoxon-Mann-Whitney test depending on variable type. The follow-up duration was defined as length of time from the date of diagnosis to the last follow-up. Overall survival (OS) was calculated from the date of definitive diagnosis to the date of death or the last follow-up. The Kaplan-Meier method was used to compare OS of patients in different groups. Factors associated with OS in HCC patients were assessed by both univariate and multivariate Cox analyses. Multivariate Cox regression analysis used a forward stepwise approach. ROC analyses were used to compare the sensitivity and specificity of survival predictions. Results are reported as hazard ratios (HR) with 95% confidence intervals (CI). Analyses were conducted with SPSS 19.0 for Windows (SPSS Inc., Chicago, IL), and a two-tailed *P* <0.05 was considered statistically significant.

## SUPPLEMENTARY MATERIALS FIGURE AND TABLE



## References

[1] Chen W, Zheng R, Baade PD, Zhang S, Zeng H, Bray F, Jemal A, Yu XQ, He J (2015). Cancer statistics in China. CA Cancer J Clin.

[2] Hanahan D, Weinberg RA (2011). Hallmarks of cancer: the next generation. Cell.

[3] Diakos CI, Charles KA, McMillan DC, Clarke SJ (2014). Cancer-related inflammation and treatment effectiveness. Lancet Oncol.

[4] Szkandera J, Gerger A, Liegl-Atzwanger B, Absenger G, Stotz M, Samonigg H, Maurer-Ertl W, Stojakovic T, Ploner F, Leithner A, Pichler M (2013). Validation of the prognostic relevance of plasma C-reactive protein levels in soft-tissue sarcoma patients. Br J Cancer.

[5] Xiao WK, Chen D, Li SQ, Fu SJ, Peng BG, Liang LJ (2014). Prognostic significance of neutrophil-lymphocyte ratio in hepatocellular carcinoma: a meta-analysis. BMC Cancer.

[6] Szkandera J, Absenger G, Liegl-Atzwanger B, Pichler M, Stotz M, Samonigg H, Glehr M, Zacherl M, Stojakovic T, Gerger A, Leithner A (2013). Elevated preoperative neutrophil/lymphocyte ratio is associated with poor prognosis in soft-tissue sarcoma patients. Br J Cancer.

[7] Szkandera J, Gerger A, Liegl-Atzwanger B, Absenger G, Stotz M, Friesenbichler J, Trajanoski S, Stojakovic T, Eberhard K, Leithner A, Pichler M (2014). The lymphocyte/monocyte ratio predicts poor clinical outcome and improves the predictive accuracy in patients with soft tissue sarcomas. Int J Cancer.

[8] Forrest LM, McMillan DC, McArdle CS, Angerson WJ, Dunlop DJ (2004). Comparison of an inflammation-based prognostic score (GPS) with performance status (ECOG) in patients receiving platinum-based chemotherapy for inoperable non-small-cell lung cancer. Br J Cancer.

[9] Stevens L, Pathak S, Nunes QM, Pandanaboyana S, Macutkiewicz C, Smart N, Smith AM (2015). Prognostic significance of pre-operative C-reactive protein and the neutrophil-lymphocyte ratio in resectable pancreatic cancer: a systematic review. HPB (Oxford).

[10] Qi Q, Zhuang L, Shen Y, Geng Y, Yu S, Chen H, Liu L, Meng Z, Wang P, Chen Z (2016). A novel systemic inflammation response index (SIRI) for predicting the survival of patients with pancreatic cancer after chemotherapy. Cancer.

[11] Jin J, Zhu P, Liao Y, Li J, Liao W, He S (2015). Elevated preoperative aspartate aminotransferase to lymphocyte ratio index as an independent prognostic factor for patients with hepatocellular carcinoma after hepatic resection. Oncotarget.

[12] Xiao GQ, Liu C, Liu DL, Yang JY, Yan LN (2013). Neutrophil-lymphocyte ratio predicts the prognosis of patients with hepatocellular carcinoma after liver transplantation. World J Gastroenterol.

[13] Candido J, Hagemann T (2013). Cancer-related inflammation. J Clin Immunol.

[14] Coussens LM, Werb Z (2002). Inflammation and cancer. Nature.

[15] Elinav E, Nowarski R, Thaiss CA, Hu B, Jin C, Flavell RA (2013). Inflammation-induced cancer: crosstalk between tumours, immune cells and microorganisms. Nat Rev Cancer.

[16] Atzpodien J, Reitz M (2008). Peripheral blood neutrophils as independent immunologic predictor of response and long-term survival upon immunotherapy in metastatic renal-cell carcinoma. Cancer Biother Radiopharm.

[17] Dings RP, Nesmelova I, Griffioen AW, Mayo KH (2003). Discovery and development of anti-angiogenic peptides: A structural link. Angiogenesis.

[18] Dunn GP, Old LJ, Schreiber RD (2004). The immunobiology of cancer immunosurveillance and immunoediting. Immunity.

[19] Kim BW, Jeon YE, Cho H, Nam EJ, Kim SW, Kim S, Kim YT, Kim JH (2012). Pre-treatment diagnosis of endometrial cancer through a combination of CA125 and multiplication of neutrophil and monocyte. J Obstet Gynaecol Res.

[20] Lee YY, Choi CH, Sung CO, Do IG, Huh S, Song T, Kim MK, Kim HJ, Kim TJ, Lee JW, Kim BG, Bae DS (2012). Prognostic value of pre-treatment circulating monocyte count in patients with cervical cancer: comparison with SCC-Ag level. Gynecol Oncol.

[21] Sasaki A, Iwashita Y, Shibata K, Matsumoto T, Ohta M, Kitano S (2006). Prognostic value of preoperative peripheral blood monocyte count in patients with hepatocellular carcinoma. Surgery.

