# Supplementary material for: Systemic inflammation response index (SIRI) predicts prognosis in hepatocellular carcinoma patients
Source: Oncotarget. 2017 Apr 5;8(21):34954–60. doi: 10.18632/oncotarget.16865 (PMC5471025; doi:10.18632/oncotarget.16865)
Supplement: Supplementary file 1 [file oncotarget-08-34954-s001.pdf]

Systemic inflammation response index (SIRI) predicts prognosis in hepatocellular carcinoma patients

Supplementary Materials

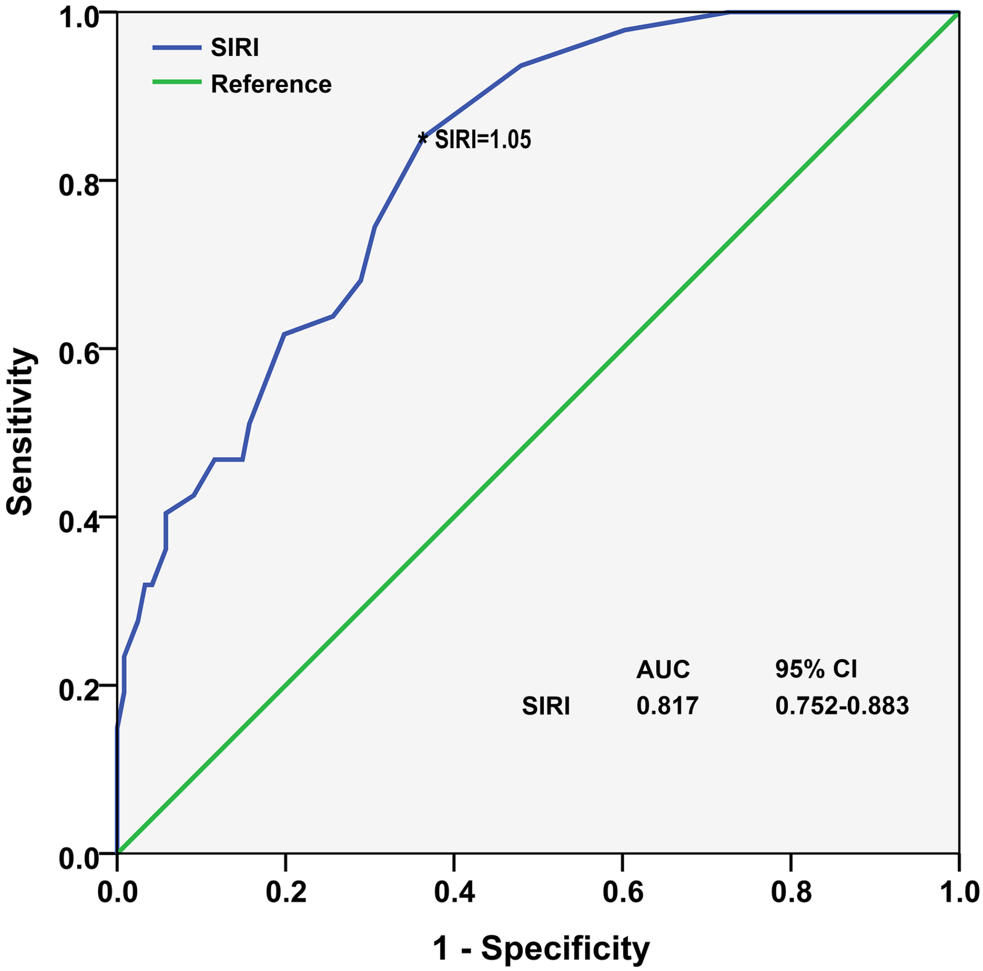

Supplementary Figure 1: The optimal cutoff point for the SIRI was determined by ROC analysis in the training cohort. The Youden index (sensitivity+specificity-1) was used to select a threshold for estimating sensitivity and specificity.

Supplementary Table 1: Baseline characteristics in different SIRI group of validation cohort

Variable	SIRI < 1.05	SIRI ≥ 1.05	*P* value
No. of patients	105	78	
Age, years, Mean ± SD	53.8 ± 11.6	53.7 ± 9.0	0.970
Gender			0.103
Male	85	70	
Female	20	8	
Hepatitis B, *n* (%)	92 (88)	64 (82)	0.295
Cirrhosis (no vs yes)	11 vs 94	9 vs 69	0.820
Child-Pugh			0.481
A	95	68	
B	10	10	
AFP			0.015
< 200 ng/ml	53	26	
≥ 200 ng/ml	52	52	
BCLC			0.022
B	65	35	
C	40	43	
TBIL (umol/L), mean ± SD	18.3 ± 14.0	18.0 ± 9.2	0.847
DBIL (umol/L), mean ± SD	6.9 ± 8.5	7.1 ± 5.1	0.843
ALT (IU/L), mean ± SD	41.4 ± 38.5	53.9 ± 60.6	0.092
AST (IU/L), mean ± SD	48.5 ± 46.9	78.0 ± 79.0	0.004
GGT (IU/L), mean ± SD	124.6 ± 124.5	232.6 ± 243.2	0.001
LDH (IU/L), mean ± SD	202.1 ± 90.4	286.1 ± 185.2	0.000
ALP (IU/L), mean ± SD	121.9 ± 79.9	174.4 ± 136.5	0.003
ALB (IU/L), mean ± SD	40.1 ± 4.4	37.8 ± 4.8	0.002
